# Early Assessment of Anxiety and Behavioral Response to Novel Swine-Origin Influenza A(H1N1)

**DOI:** 10.1371/journal.pone.0008032

**Published:** 2009-12-03

**Authors:** James Holland Jones, Marcel Salathé

**Affiliations:** 1 Department of Anthropology, Stanford University, Stanford, California, United States of America; 2 Department of Biological Sciences, Stanford University, Stanford, California, United States of America; 3 Woods Institute for the Environment, Stanford University, Stanford, California, United States of America; Swiss Paraplegic Research, Switzerland

## Abstract

**Background:**

Since late April, 2009, a novel influenza virus A (H1N1), generally referred to as the “swine flu,” has spread around the globe and infected hundreds of thousands of people. During the first few days after the initial outbreak in Mexico, extensive media coverage together with a high degree of uncertainty about the transmissibility and mortality rate associated with the virus caused widespread concern in the population. The spread of an infectious disease can be strongly influenced by behavioral changes (e.g., social distancing) during the early phase of an epidemic, but data on risk perception and behavioral response to a novel virus is usually collected with a substantial delay or after an epidemic has run its course.

**Methodology/Principal Findings:**

Here, we report the results from an online survey that gathered data (*n* = 6,249) about risk perception of the Influenza A(H1N1) outbreak during the first few days of widespread media coverage (April 28 - May 5, 2009). We find that after an initially high level of concern, levels of anxiety waned along with the perception of the virus as an immediate threat. Overall, our data provide evidence that emotional status mediates behavioral response. Intriguingly, principal component analysis revealed strong clustering of anxiety about swine flu, bird flu and terrorism. All three of these threats receive a great deal of media attention and their fundamental uncertainty is likely to generate an inordinate amount of fear vis-a-vis their actual threat.

**Conclusions/Significance:**

Our results suggest that respondents' behavior varies in predictable ways. Of particular interest, we find that affective variables, such as self-reported anxiety over the epidemic, mediate the likelihood that respondents will engage in protective behavior. Understanding how protective behavior such as social distancing varies and the specific factors that mediate it may help with the design of epidemic control strategies.

## Introduction

An ongoing outbreak of novel influenza A(H1N1), colloquially referred to as “swine flu,” has caused over 200,000 confirmed cases (as of 28 August 2009 [1]). Because of under-reporting, the actual number of people infected is substantially larger, particularly considering that many countries have ceased testing for A(H1N1) [1]. As human-to-human transmission became widespread in at least one region of the world, WHO rapidly declared the outbreak an imminent pandemic [2] and with widespread human infection, WHO declared a phase 6 pandemic on 11 June 2009, where it remains at the time of submission [3]. The virus appears to have a higher reproduction number and somewhat higher case fatality ratio than recent seasonal influenza viruses [4], [5], and has certainly caused great concern in the population, fueled by both extensive media coverage and an initially high level of uncertainty about mortality rates and transmissibility of the virus.

Mathematical and computational models are useful for predicting the fate of an epidemic, and while such models have become increasingly complex and realistic in recent times, a key ingredient is often ignored: human behavioral responses to the threat and/or presence of a disease [6]. How people assess risk of infection and how such risk assessment drives behavioral change is of great interest as individual social distancing can greatly affect the spread of an epidemic [7], [8], [9]. While the effect of behavioral change in response to perceived health threats on the spread of infectious diseases has been investigated theoretically for some time, particularly in the context of sexually transmitted diseases [8], recent work has started addressing the problem in a broader context that is also applicable to the spread of influenza [6], [7]. This work has a strong, though as yet under-explored relationship to work on risk perception and health threats [10], [11], [12].

Data on risk perception and behavioral response in the general population have rarely been collected right from the outset of an epidemic. Instead, they are usually gathered with a substantial delay in the case of influenza A(H1N1) [13], after the epidemic has run its course, as in the case of SARS [9], or before sustained human to human transmission is established, as in the case of highly pathogenic avian influenza A(H5N1) [14]. However, the feasibility of halting or mitigating the spread of an infectious disease is highest during the very early phases of an outbreak, and thus data on behavioral response during this time would provide valuable information for public health policy and research. Here, we report the results from an online survey that gathered data (*n* = 6,249) about risk perception of the outbreak during the first few days of widespread media coverage (April 29 – May 5, 2009) of the emergence of novel swine-origin Influenza A(H1N1).

## Methods

### Ethics Statement

The research presented here was approved by the Stanford University Non-Medical Human Subjects Institutional Review Board on 28 April 2009 (Protocol #16782).

### The Sample

We fielded in internet-based survey starting on 29 April 2009 using Opinio survey software [15]. The URL for the survey is (https://opinio.stanford.edu/opinio/s?s=1403). The sampling universe for this study was adults 18 and older with access to a networked computer. The initial seed for the sample was generated using social networking software, and a request sent to a standing subject pool comprised of Stanford alumni and social science students at a nearby community college maintained by the Institute for Research in the Social Sciences at Stanford University. The survey was picked up by a variety of internet media sources including several science general media blogs. Directly following publicity in these blogs, we received the most responses. Table 1 summarizes the sample.

**Table 1:** Summary statistics for survey sample.

Measure	Value
Mean Age	37.6 (sd = 12.7, range: 18–93)
Fraction Female	0.466
Mean Household Size	2.43 (sd = 1.31)
Number with HS Degree or Less (Fraction)	250 (0.04)
Number with Some College (Fraction)	1131 (0.181)
Number with College Degree (Fraction)	2131 (0.341)
Number with More than College Degree (Fraction)	2712 (0.434)
Number of US Respondents (Fraction)	4318 (0.691)
Total Respondents	6,249

### Definition of Variables

The survey was designed to get a rapid assessment of respondents' affective state, sources of information on the emerging pandemic, and the behaviors undertaken for protection while minimizing respondent burden. As such, it included only 17 questions.

Questions probing subjective assessment of risk perception, level of anxiety, and ability to avoid flu infection were asked on a 9 point ordinal scale with anchors at the extrema (“very high”, “very low”) and the center (“intermediate”). Subjective emotional status (i.e., respondents' affective state regarding the epidemic) was anchored by the terms “very calm” through “intermediate” to “very anxious.” Comparative questions of subjective risk perception for eight health threats were asked using a five-point ordinal scale with anchors at all points: “very low,” “low,” “intermediate,” “high,” and “very high.” Questions regarding media (both respondents' frequency of getting information from a particular source and their judgment of each source's accuracy) were asked on a five point ordinal scale with anchors at all points (“very often/accurate” to “never/almost certainly inaccurate”). Respondents' knowledge of swine flu was assessed with a series of six True/False questions. Respondents gave free-text responses to questions about their current age, the number of people currently living in their household (including themselves) and their zip code if they currently live in the United States. Respondents who reported not currently living in the United States were asked to report their current country of residence in a free-text field. A screen-shot of the full survey instrument is included in the Supplementary Material.

For our analysis of participants' response to the threat of swine flu, we use a variable we call “survey day.” The survey went online in the evening of 28 April, Pacific time, so we combined responses from 28 and 29 April into a single day. This combined day of 28–29 April represents survey day 1.

Subjects were asked to state the number of contacts in the past 24 hours. Contacts were defined by close physical contact as operationalized by a face to face conversation of more than two words in the presence of another individual or physical exposure involving skin contact such as a handshake, hug, or contact during sporting activities. Respondents were provided five ordered categories: less than 5, 5–10, 11–20, 21–50, 51–100, more than 100. Handcock and Jones [16] discuss the phenomenon of heaping and related problems for statistical inference in answering epidemiological questions regarding contact number. Structuring responses within broad ordinal categories avoids many of the pitfalls of contact-heaping encountered in epidemiological investigations.

### Protection Index

To measure the response in epidemiologically relevant behavior to information on the potential pandemic, we asked a series of questions about protective actions taken by the respondents. In the survey, we asked: “Given the current status of the epidemic, which of the following precautionary actions will you take?”

Avoid people who cough/sneeze

Avoid large gatherings of people

Wash hands more often

Avoid people who are in contact with infected people

Avoid public transportation

Avoid school/work

Avoid travel to infected areas

Use disinfectant

Wear a mask

Not all of these behaviors are necessarily effective or recommended protective measures (e.g., wearing a mask), but our aim was to gauge people's attempt at self protection so even non-efficacious behavioral change is interesting in that it indicates willingness to act on the part of the respondent.

We constructed an index of protective behavior by summing the answers to the questions regarding actions taken to avoid influenza infection. The index ranged from 0–9, with larger values indicating more protective measures taken. Using a binomial GLM with canonical logit-link, we modeled the protection index as a function of covariates. Our primary interest was the possible mediating effect of affective variables on action taken to protect against swine flu infection. To evaluate the hypothesis that respondents' affective state (subjective anxiety, fatalism about infection) predicts protective measures, we include in the model demographic (age, gender), epidemiological (household size, number of contacts, survey day), and media (source of information on the outbreak) conditioning variables. For the media variables, we constructed dummies with a value of 1 corresponding to answers of “very often” or “often” and a value of 0 for all other responses to the question of “How often do you use the following sources to get information about swine flu?” With such a large number of conditioning variables, in addition to the affective variables of greatest interest, there is a distinct danger of over-fitting the GLM. To address this problem, we used likelihood-based model selection [17] to search the model space set up by our conditioning variables [18].

Of the nine protective behaviors, increased hand-washing is both the simplest and probably most effective at curbing transmission. As such, it is strongly advocated in infection control educational material [19]. In addition to our tests for predictors of the protection index, we therefore also tested the effect of measured covariates on the odds of increased hand washing using a binomial GLM again with canonical logit-link.

### Perceived Risk Clustering

A concern regarding the relationship of people's self-reported anxiety and their protective behavior is that some people might generally be more anxious regarding health. We probed general anxiety by asking about respondents' anxiety with regard to a number of infectious, chronic, and violent threats to health. We asked a series of questions probing respondents' perceived subjective risk on a 5-point scale for a variety of health threats, including other infectious diseases (A(H5N1) “bird flu”, seasonal flu, HIV/AIDS), chronic diseases (heart disease, diabetes, cancer), and violence (unintentional accidents, terrorism). We calculated the correlation matrix for answers to these threat questions and used Principal Components Analysis (PCA) to explore potential structure in the responses to different categories of threat [20].

## Results

We begin by presenting descriptive results of the survey and follow with our primary analytical questions from the survey, namely, testing the hypothesis that respondents' affective state mediates their protective action.

We gathered 6,249 responses from 28 April to 5 May 2009. Table 1 presents descriptive statistics of the sample.

**Figure 1:**
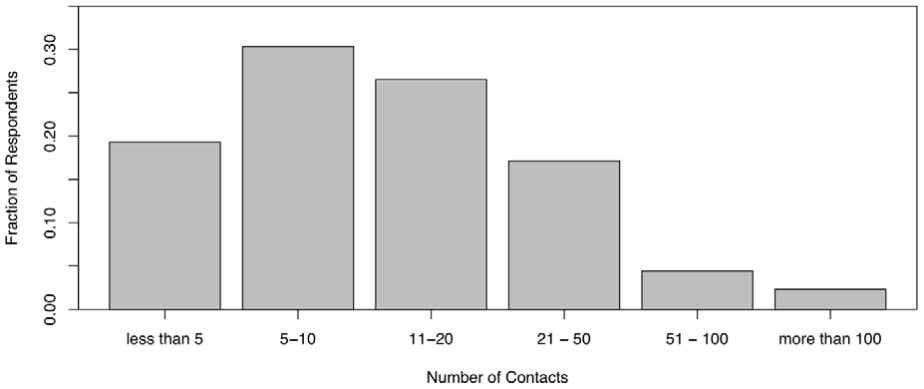
Frequencies of the categories of respondents' contacts outside the home.

Figure 1 presents the distributions of respondents' contacts within the 24 hours prior to taking the survey.

**Figure 2:**
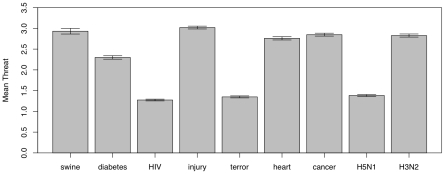
Means for the perceived threat levels for different sources of risk. Bars show Bonferroni-corrected 95% confidence intervals. Codes: 1 = no risk, 5 = very high risk; “swine” = Novel Swine-Origin Influenza A(H1N1), “diabetes” = Diabetes mellitus, “HIV” = HIV/AIDS, “injury” = Unintentional injury, “terror” = Terrorism, “heart” = Heart Disease, “cancer” = Cancer, “H5N1” = Bird Flu, “H3N2” = Seasonal Flu.

Figure 2 presents the means of the subjective threats. Swine flu had a mean second only to injury, and the highest among the infectious sources of threat. The mean of perceived threat from swine flu fell above the Bonferroni-corrected 95% confidence interval for all other threats but unintentional injury.

**Figure 3:**
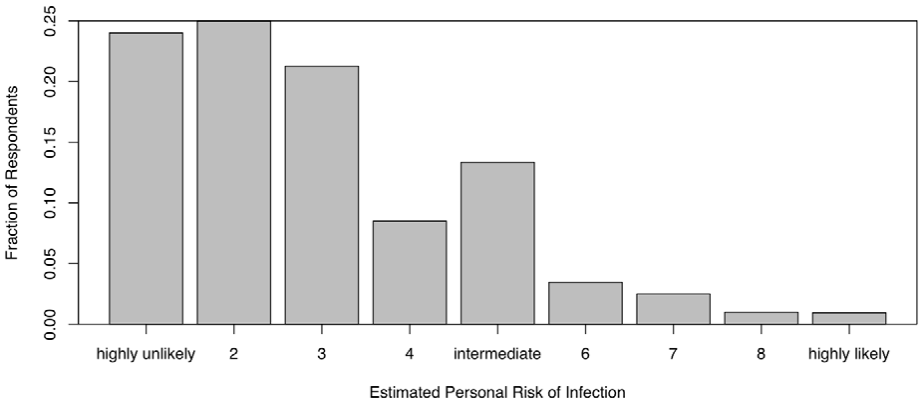
Frequency distribution of personal risk perceptions. While most respondents rate their personal risk as low, note the pronounced second mode at the intermediate level of risk perception.

**Figure 4:**
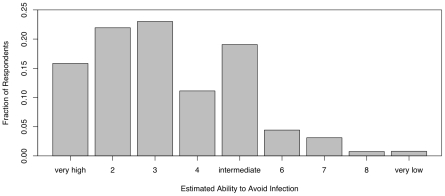
Frequency distribution of personal empowerment. Again, while most respondents list a high level of personal empowerment, there is a decided second mode at the intermediate level (1 = “very high: I feel confident I can avoid infection”, 5 = “intermediate”, 9 = “very low: I feel I will not be able to avoid infection”).

Figure 3 presents the frequency distribution of perceived personal risk. There is a notable bimodality to this plot. This apparent bimodality is not simply attributable to sampling error since the difference between the responses = 4 vs. those  = 5 vs. those  = 6 is in excess of 300. Further analysis using finite mixture models [21] provides strong statistical support for the reality of the bimodal pattern (results not shown). While the majority of respondents felt that their personal risk was low, there is a second mode rating their risk as intermediate ( = 5). This same bimodal pattern can be seen in the frequency distribution of personal empowerment (i.e., ability to avoid infection) shown in figure 4. While most respondents indicate that they are confident they can avoid infection, a substantial second mode appears at the intermediate value.

**Figure 5:**
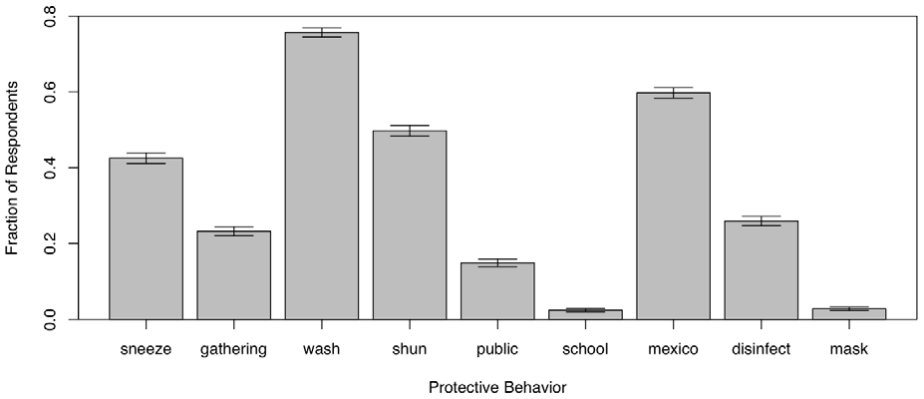
Frequency distribution of the protection measures undertaken by respondents. (“sneeze” = avoid people sneezing or coughing, “gathering” = avoid large gatherings, “wash” = wash hands more frequently, “shun” = avoid people perceived to be sick and potentially infectious, “public” = avoid public places, “school” = stay home from school or work, “Mexico” = avoid travel to affected foreign countries or states, “disinfectant” = use alcohol-based disinfectant, “mask” = wear a protective mask).

Figure 5 shows the frequency distribution of protective behaviors. We can see that nearly 80% of respondents report washing hands more frequently, while very few avoid work or school or wear protective masks.

**Figure 6:**
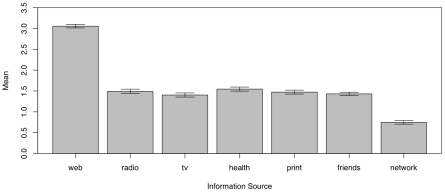
Mean values of sources of information on swine flu cited by respondents. Bars show Bonferroni-corrected 95% confidence intervals. Codes: 1 = never use as source of information, 5 = very frequently use as a source of information.

Figure 6 shows the means for respondents' information sources. Not surprisingly, the most common source of information reported was the Internet. Again, mean values are plotted with their 95% Bonferroni-corrected confidence intervals. With the exception of social-networking tools (e.g., Facebook, Twitter), all other media sources are statistically indistinguishable from each other, with the social-networking tools being used significantly less.

The results of the model for the protection index show a number of robust trends (table 2). In particular, we find that age increases and male gender decreases the protection index. Receiving a large amount of information from the internet, television, and health officials all increase the protection index while receiving large amounts of information from print media, friends, or social networking media has no effect. The number of household members has no discernible effect, though the number of contacts outside the home does. For the ordered factor “contacts,” the first category (<5 contacts in the past 24 hours) is the reference category. Interestingly, relative to respondents with the fewest number of contacts, all other contact categories have reduced protection indices, indicating that people with fewer contacts take more protective actions. Not surprisingly, residence in Mexico has a large positive effect, while residence in Canada or Europe decreased the index. The day that the survey was taken (29 April = 1) had a negative effect on the index, indicating that respondents took less protective action as the epidemic proceeded. Respondents' reported subjective anxiety has a substantial impact on the index with high anxiety increasing protection, supporting our hypothesis that affective state mediates protective behavior.

**Table 2:** Results of the binomial GLM for the protection index.

Variable	Estimate	Std. Error	z-score	p-value
(Intercept)	−1.2559	0.0517	−24.32	<0.001
Age	0.0060	0.0008	7.86	<0.001
Male Gender	−0.1499	0.0189	−7.92	<0.001
Household Size	0.0437	0.0192	2.28	0.0226
5–10 contacts	−0.1454	0.0267	−5.44	<0.001
11–20 contacts	−0.2506	0.0279	−9.00	<0.001
21–50 contacts	−0.2502	0.0310	−8.07	<0.001
51–100 contacts	−0.2393	0.0496	−4.82	<0.001
>100 contacts	−0.3758	0.0673	−5.58	<0.001
Survey Day	−0.0406	0.0074	−5.48	<0.001
Info: Internet	0.1899	0.0219	8.68	<0.001
Info: Radio	0.0754	0.0243	3.11	0.0019
Info: TV	0.1496	0.0237	6.32	<0.001
Info: Health Official	0.1520	0.0225	6.77	<0.001
Info: Print	−0.0506	0.0242	−2.09	0.0363
Europe	−0.2477	0.0309	−8.01	<0.001
Mexico	0.6507	0.1354	4.81	<0.001
Canada	−0.1176	0.0392	−3.00	0.0027
Risk	0.0514	0.0065	7.93	<0.001
Confidence	−0.0439	0.0066	−6.61	<0.001
Anxiety	0.1856	0.0067	27.73	<0.001

Variables of the form “Info: XYZ” refer to media source dummy variables that are 1 if the respondent gets information from that source very often or often and zero otherwise. See Methods section for definition of other variables. For the ordered factor, contacts,<5 is the reference category.

Increased hand-washing showed similar trends to the model for the protection index (table 3). Male gender decreases while age and survey day increase the odds of increasing hand-washing. Receiving a large amount of information from the internet, radio, television, and health officials increase, while living in Europe or Australia/New Zealand decrease the odds. As with the overall protection index, perception of risk and subjective anxiety significantly increase the odds of increased hand-washing modestly.

**Table 3:** Results of the binomial GLM for increased hand-washing.

Variable	Estimate	Std. Error	z value	p-value
(Intercept)	−0.4303	0.1518	−2.84	0.0046
Age	0.0115	0.0027	4.25	<0.001
Male Gender	−0.5118	0.0662	−7.73	<0.001
Survey Day	0.0571	0.0254	2.24	0.0249
Info: Internet	0.4089	0.0696	5.87	<0.001
Info: Radio	0.5064	0.0962	5.27	<0.001
Info: TV	0.2502	0.0905	2.76	0.0057
Info: Health Official	0.4564	0.0897	5.09	<0.001
Europe	−1.1163	0.0895	−12.47	<0.001
Oz	−0.7284	0.1810	−4.02	<0.001
Risk	0.1040	0.0223	4.66	<0.001
Anxiety	0.3028	0.0280	10.83	<0.001

Variable definitions as in table 2.

### Changes in Behavior

A key epidemiological question is how people's affective status and protective behaviors undertaken change as the epidemic proceeds. To develop a measure for this, we cross-tabulated individual values of the protection index and affective status by survey day. Pearson's chi-square test for independence of both tables was strongly significant (affective: χ^2^ = 135.6, *df* = 48, *p*<0.001; protection: χ^2^ = 113.1, *df* = 54, p<0.001), indicating substantial departure of cells from the expected values. To visualize the pattern of departure from the expected values, we calculated an expected tables taken as the cross-product of the marginals of the observed table normalized by the grand sum. We combined rows of these tables to simplify the presentation, plotting the difference between observed and expected tables for a high, medium and low emotional status/protection index respectively. For example, a value of −51 on the calm affective status on day one means that there were 51 fewer responses in the calm categories than would be expected by the overall marginal distribution of responses across all days.

**Figure 7:**
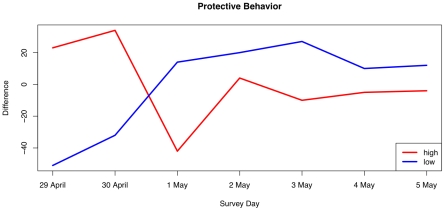
Changes in difference between observed and expected values of the protection index over the seven days of the survey. The pattern is significantly different from the expected pattern based on marginal frequencies (χ^2^ = 113.1, *df* = 54, p<0.001).

**Figure 8:**
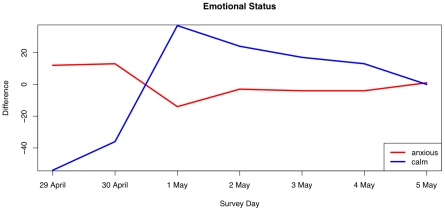
Changes in difference between observed and expected values of the emotional status over the seven days of the survey. The pattern is significantly different from the expected pattern based on marginal frequencies (χ^2^ = 135.6, *df* = 48, *p*<0.001).

In figures 7 and 8, we plot the change in respondent's protective behavior and emotional status over the first week of the survey. The lines represent the differences between observed and expected frequencies of responses for the 9-point scale simplified to three levels each. We see that by day three of the survey (May 1st), the relative number of people reporting a calm emotional state was very high, while the number of people reporting high values of the protective index declined dramatically. We interpret these results to indicate that people's response to a potential pandemic is quite sensitive to media reports.

In general, individuals' survey responses to perceived risk for the eight health threats were only moderately correlated, with pairwise correlations typically well under 0.5. PCA did not reveal that a substantially reduced number of dimensions explained these correlated data – six principal components were required to explain 85% of the variance. Nonetheless, some intriguing PC loadings revealed themselves. In particular, the second PC, which explained 15.6% of the variance in the data, showed strong positive correlations with swine flu (r = 0.516), bird flu (r = 0.530), and terrorism (r = 0.467). All three of these threats receive a great deal of media attention and their fundamental uncertainty are likely to generate an inordinate amount of fear vis-a-vis their actual threat [22].

## Discussion

Our results indicate that respondents' behavior varies systematically with covariates from demographic, epidemiological, media, and affective domains. People's anxiety about swine flu and the preventative actions they took to avoid infection declined as the perceived gravity of the novel outbreak waned. Overall, subjective risk perception was low and people's belief in their ability to avoid infection was high. Both of these distributions nonetheless showed a marked bimodality, with a large proportion of respondents indicating a higher subjective risk and more protective actions taken than the majority (Figures 3–4).

The results of our statistical modeling suggest that respondents' deployment of protective behavior is mediated by their subjective anxiety level, controlling for demographic, epidemiological, and geographic variables. There is good and bad news in this result. The literature on risk perception and public health shows that there is generally a very weak correlation between people's anxiety over a particular risk and the probability of death or disability arising from that risk [11], [12]. Overall, it is unclear whether anxiety over perceived risk will lead to efficacious protective behaviors [10]. This said, by far the most common protective behavior reported in our survey was increased hand-washing, which has been shown to be effective at removing Influenza A(H1N1) virus from subjects' hands [23] and is promoted by CDC and other health organizations as an effective infection control intervention [19].

One curious result from the model for the protection index is that people with the fewest contacts have marginally higher protection indices. There are two potential explanations for this finding: (1) individuals with small social support networks (and consequently, few contacts outside the home) are more anxious, making it more likely that they will take greater protective actions or (2) people concerned about infection and taking relatively many protective actions also reduce the number of contacts they have and therefore had a small number of contacts in the past 24 hours. The first explanation is consistent with work in social epidemiology on the role of social networks in mediating infection risk [24], [25] Because of the nature of the sample, we are unable to evaluate the direction of causality that leads to this result. Nonetheless, it remains an intriguing hypothesis.

Many questions about this Novel Swine-Origin influenza A(H1N1) virus remain. Of particular concern is the possibility that the virus could mutate during the flu season in the southern hemisphere and selection could drive it to become more virulent as it returns to the northern hemisphere in Autumn. Worryingly, such a pattern of multiple waves with an increased proportion of the total influenza-associated mortality burden has been reported for all three past influenza pandemics [26], [27]. Finding a means to simultaneously communicate to the public the structural uncertainty inherent in projecting pandemics and the seriousness of a pandemic after the media frenzy about its emergence has died down remains a major challenge to public health.

Pharmaceutical interventions such as vaccines and antiviral drugs may form a strong line of defense, but the efficacy of such measures remains unclear and depends on the particular biology of a given pathogen. This is exacerbated by people's reluctance to be vaccinated [28]. With more than 300 infectious diseases emerging within less than a century [29], the threat of pandemic influenza is only the latest out of many public health threats posed by infectious diseases in a globalized world. Unlike pharmaceutical interventions, non-pharmaceutical interventions like social distancing may be effective in halting or at least mitigating an epidemic independent of the specific biology of a pathogen, and thus provide a reliable set of strategies to combat infectious diseases that warrant further attention [30]. Our results that people do not rely on social networking tools to the extent that they rely on other media may have implications for CDC strategies for spreading public health information via social media [19]. In particular, public health messages spread via social media will need to backed up by information spread via more traditional channels, which respondents list as being common sources of trusted information on the outbreak.

Our study is subject to a number of weaknesses. The advantage of our internet-based sampling strategy is the ability to quickly deploy a survey and thereby track responses in near real-time. The clear disadvantage of this strategy is a sacrifice of population representativeness. Despite its general availability on the internet, our sample shows a pronounced bias for highly-educated respondents living in the Western United States. These biases clearly limit the generality of our results, but the large number of respondents filing out the survey as information on the potential pandemic changed nonetheless provides a uniquely valuable data source. Within one week (the cutoff point for the current analysis), we had a sample of 6,249 responses. In contrast, the telephone-based study of Rubin et al. [13] employed a random-digit-dial sampling design, allowing a more representative sample of the general UK population, but their sample was only 997 respondents and the survey was undertaken after media attention had abated, beginning 8 May 2009. Nonetheless, the results reported in this paper are largely congruent with our own results and we see the studies as strongly complementary.

Our respondents began filling out the survey on the day that WHO upgraded the pandemic threat category of the H1N1 outbreak from 4 to 5 and spans the week in which the number of WHO-confirmed cases increased tenfold. While our sampling design is subject to many of the usual criticisms of internet-based surveys and is not necessarily representative of the general population, the unparalleled immediacy, longitudinal nature, and the large number of respondents it contains make our data set unique and scientifically important for the study of the spread of information and distribution of risk perception and behavioral change during the most uncertain time (i.e. the initial phase) of an epidemic of a virus novel to the human population.
